# Supplementary material for: Experiences of hospice dementia care: A qualitative study of bereaved carers and hospice clinicians
Source: PLoS One. 2023 Nov 6;18(11):e0286493. doi: 10.1371/journal.pone.0286493 (PMC10627455; doi:10.1371/journal.pone.0286493)
Supplement: Attachment — Submitted filename: Response to reviewers.docx [file pone.0286493.s001.docx]

Table for reviewers.

| **Editor** | | |
| --- | --- | --- |
| **Where in the manuscript** | **Reviewers’ comments** | **Our response to reviewers** |
|  | 1. It is important that you include a cover letter with your manuscript. Please ensure that this letter is addressed specifically to PLoS ONE. Please also include  A) why this manuscript is suitable for publication in PLoS ONE.  B) how does your paper provide a worthwhile addition to the scientific literature?  C) how does your paper relate to previously published work?  D) which types of scientists do you believe will be most interested in your study? | This has now been drafted and uploaded. |
|  | 2. Please upload a Response to Reviewers letter which should include a point by point response to each of the points made by the Editor and / or Reviewers. (This should be uploaded as a 'Response to Reviewers' file type.) Please follow this link for more information: <http://blogs.PLOS.org/everyone/2011/05/10/how-to-submit-your-revised-manuscript/> | This has been uploaded |
|  | 3. Please include a figure label and title for Figure 1 in your main manuscript. | The label has been added in the manuscript (Page 16 Line 735). Please note that the actual figure has been uploaded as a separate file. |
|  | 4. It appears that your ORCiD iD has not been validated in your Editorial Manager account and we are unable to proceed until that step is complete.  To validate your ORCiD iD in Editorial Manager, please follow the steps below:  A. In your Editorial Manager account, please go to ‘Update my Information’ (in the upper left-hand corner of the main menu), and click on the Fetch/Validate link next to the ORCiD field.  B. This link will take you to the ORCiD site and allow you to create a new iD or authenticate a pre-existing iD in Editorial Manager.  For additional instructions, please watch the following video for a step-by-step demonstration: <https://www.youtube.com/watch?v=_xcclfuvtxQ> | It appears that now the ORCID is linked to authors’ login. |
|  | 5. Please note that funding information should not appear in the Acknowledgments section or other areas of your manuscript. We will only publish funding information present in the Funding Statement section of the online submission form. Please remove any funding-related text from the manuscript. | This has now been deleted from manuscript |
|  | 6. Thank you for stating the following financial disclosure:  “Yes. Data collection occurred in the premises (hospice) where the funder is located.”  Please state what role the funders took in the study. If the funders had no role, please state: "The funders had no role in study design, data collection and analysis, decision to publish, or preparation of the manuscript."  If this statement is not correct you must amend it as needed.  Please include this amended Role of Funder statement in your cover letter; we will change the online submission form on your behalf. | We have now changed this. |
|  | 7. We note you state the data underlying the results presented in the study are available from the corresponding author  Please note, in line with our goal of ensuring long-term data availability to all interested researchers, PLOS’ Data Policy states that authors cannot be the sole named individuals responsible for ensuring data access (http://journals.plos.org/plosone/s/data-availability#loc-acceptable-data-sharing-methods). | We have now reported the REC committee for data sharing requests. |
| Before we proceed with your manuscript, please address the following prompts:  a) If there are ethical or legal restrictions on sharing a de-identified data set, please explain them in detail (e.g., data contain potentially identifying or sensitive patient information, data are owned by a third-party organization, etc.) and who has imposed them (e.g., a Research Ethics Committee or Institutional Review Board, etc.). Please also provide non-author contact information for a data access committee, ethics committee, or other institutional body to which data requests may be sent.  We will update your Data Availability statement on your behalf to reflect the information you provide. |  | We have now explained this and reported the REC committee for data sharing requests. |
| ***General comments*** | a) If there are ethical or legal restrictions on sharing a de-identified data set, please explain them in detail (e.g., data contain potentially sensitive information, data are owned by a third-party organization, etc.) and who has imposed them (e.g., an ethics committee). Please also provide contact information for a data access committee, ethics committee, or other institutional body to which data requests may be sent. | We have now explained that: *Data contain potentially sensitive information as only one hospice acted as recruitment site. The data underlying the results presented in the study are available from the corresponding author (ab2984@medschl.cam.ac.uk).* |
|  | b) If there are no restrictions, please upload the minimal anonymized data set necessary to replicate your study findings as either Supporting Information files or to a stable, public repository and provide us with the relevant URLs, DOIs, or accession numbers. For a list of acceptable repositories, please see http://journals.plos.org/plosone/s/data-availability#loc-recommended-repositories. | Please see response above. |
|  | 4. PLOS requires an ORCID iD for the corresponding author in Editorial Manager on papers submitted after December 6th, 2016. Please ensure that you have an ORCID iD and that it is validated in Editorial Manager. To do this, go to ‘Update my Information’ (in the upper left-hand corner of the main menu), and click on the Fetch/Validate link next to the ORCID field. This will take you to the ORCID site and allow you to create a new iD or authenticate a pre-existing iD in Editorial Manager. Please see the following video for instructions on linking an ORCID iD to your Editorial Manager account: <https://www.youtube.com/watch?v=_xcclfuvtxQ> | I was not able to link the ORCID number on the system. Please could you link my ORCID ID below?  Thank you.  Orcid: https://orcid.org/0000-0002-2374-3427 |
|  | 5. Your ethics statement should only appear in the Methods section of your manuscript. If your ethics statement is written in any section besides the Methods, please move it to the Methods section and delete it from any other section. Please ensure that your ethics statement is included in your manuscript, as the ethics statement entered into the online submission form will not be published alongside your manuscript. | We have now added this in the methods section (Page 5 Lines 178-180). |
|  | -Please, remember to put the continuous line numbering on the entire manuscript and the manuscript should be well spaced. This will enable ease during review and editing as it allows the reviewers and Editors to easily point out errors and corrections needed in the manuscript. | We have now inserted continuous numbering as per suggestion. |
| ***Abstract*** | Results  -This part of the abstract should be under methods since it is reflecting the number and type of participants that were recruited and interviewed “A total of 12 participants were interviewed from one service in the Northwest region in the UK. All were female and white British.” | Thank you for your comment, however we feel that this information belongs to the results section. |
|  | -Clearly state the generated themes and the major challenges encountered in your study and delete this statement “Themes identified clustered around the experience of pre-access to hospice care, role and responsibility of staff and carers within hospice, and the challenges of delivering care during the last days of life for the person with dementia.” | We feel we have now addressed this. (Page 3 Lines 86-87). |
|  | - Conclusion   State clearly what need to done to improve the services | We have now addressed this (Page 3 Lines 90-93). |
| ***Background*** | -I released that the title background/introduction is missing in the manuscript. | We have now added this (Page 4 Line 121). |
|  | -The research question should not be a heading after the background but part of the background information. “Primary Research Question: What is the experience of end-of-life hospice care for bereaved carers of people with dementia?” | We have now addressed this (Page 5 Lines 171-172). |
|  | -Please, ensure that the objective or aim of the study is the last sentence in the last paragraph of your background/introduction | We have now addressed this in the text (Page 6 Lines 240-242). |
| ***Methods*** | -Shouldn’t the participants be given full information on the study before signing the consent form? Why giving them after the interview? As stated below in your manuscript:  “All participants who agreed to take part in the qualitative interviews were required to sign a consent form and at the end of the interviews they were provided with a debriefing form summarising the aims of the study and how to address any issues (if any) that could occur during or after the study. Bereaved carers were only approached if a minimum of” | We have explained this more clearly in the text, All participants were asked to sign a consent form before the interview could take place, (Page 5 Lines 240-242). |
|  | -Take a paragraph each to express the eligibility criteria for carers and hospice clinical personnel instead of using bullet points. | We have now addressed this (Page 6 Lines 250-261). |
|  | -The statement for data storage should be under data availability and not part of the methods. | We have now removed the following form the text as per suggestion: *The main author was responsible for all data activities for the research project, including but not limited to: data capture, data quality, data storage, and data sharing with transcription agency which was one of the University of Manchester approved supplier. Interview transcripts and encrypted audio recordings of interviews were saved onto the University of Manchester Research Data*  *Storage Service (Isilon). The audio recordings were transferred as soon as possible (and after the audio file is checked for quality) into the University Data Storage Service and erased from the temporary storage device (audio recording device). Audio recordings were encrypted to AES 256 standard when not in use in the Data Storage Service (UoM).* |
|  | -What were the methods you used to eliminate or minimize bias? Inductive coding and or reflexivity?.... | We have used inductive coding and we have clearly termed the analytic method (Page 7 Line 279). |
|  | -What type of coding method did you use? From the study I realized that you used inductive method of coding. State it clearly in your methods. | Yes we used inductive coding and we have now included it in the text (Page 7 Line 279). |
|  | -Nothing was mentioned with regards to “data saturation point”. Did you at any point of the interview achieve data saturation? Please state whether or not this was achieved as part of the methods. If data saturation was not achieved during the interview, kindly state it as part of the limitation of your study. | Yes we added in the text more prominently that we based our sample size on the Sample size on conceptual density (i.e., gathering data until a sufficient depth of understanding was reached), which was agreed upon by all study authors. (Page 7 Lines 265-267). |
| ***Results*** | -The statement below should be part of the methods involved in recruitment of participants and should not be part of the results. Please, insert this section under methods in the appropriate location:  “Semi-structured interviews were held with 12 participants (seven clinical hospice staff and five family carers, all were female and white British) form one service in the Northwest region. Two staff were medically trained in general practice, three were nurses with one being a palliative care specialist, two were health care assistants. The interviews lasted on average 38.30 minutes.” | We appreciate the comments but all authors felt that this would be more appropriate in the results section. |
|  | -Please, do include a table that will clearly indicate the THEMES, SUBTHEMES, CODES GENERATED and REPEATITION OF CODES by participants for more clarity on the data analysis and understanding of the data at a glance. This table should be in the result section and modified as indicated (Table 1). Participant account may not be necessary in the Table, it has already been stated under the various themes and subthemes in the result section. | We engaged in discussion with our PPEI and they felt that reporting more quotes in the table is useful for the reader, so we have decided to maintain the format of the table and its content as it was when first submitted. |
|  | -Covid should be written as COVID all through the manuscript please. | We could only find the term in Table 1 and we corrected it. |
|  | -Do you mean in the early phase of the disease (dementia)? “when they're earlier in their disease”, you may need to rephrase the statement for clarity.  Please paraphrase the statement for clarity - “So I think about half patients come in for symptom control and then probably the other half of patients come in for end-of-life care.” Do you mean half of the incoming patients? | We have now clarified this (Page 13 lines 542-543; Page 13 Line 571). |
|  | -I do understand that the translations and transcriptions were done verbatim but some of the transcriptions need to be properly translated for clarity and at the same time making sure that you do not alter the meaning of what the participant said. For example the statement from one of the participants below located in pages 12 and 13 of the manuscript:  “…I think generally in that case they are with us for end of life. I think some patients would come to us with maybe you know, co-morbidities, dementia and a cancer for example and if we, through being with us we achieve or they achieve better symptom control and you know, there’s an opportunity for them to go home, because they’re not imminently end of life 13 or go, you know, be discharged really, then obviously we would aim to do that if it, if that’s in, you know, in keeping with their wishes, either whether that is to home or to a nursing home and then obviously when planning their discharge we would have to take the dementia diagnosis and the symptoms and the issues that go with that into consideration when planning their discharge.’ (Staff 1 – clinical specialty in general practice).” | We seem to have now corrected this. |
| ***Discussion*** | -Please, delete all the statements highlighted below because the statement is about the methods and partly results. Just discuss your findings straight ahead. The statements have been dealt with earlier on in the manuscript and there is no need for repetitions. Do not put Figures in discussion please.  “This was a qualitative study exploring the experience of bereaved carers and hospice clinical personnel of hospice dementia care. We employed thematic analysis for theme categorization and data interpretation. From the available data we found themes around the experience of pre-access to hospice care, during care delivery and around the bereavement support available to carers soon after the death of the person with dementia. The data allowed us to have a better picture of hospice care pathway that is currently being offered to people with dementia in one of the settings in the Northwest region in England (Figure 1)” | We have now removed the text from the discussion and moved it to methods where we describe the service (Pages 5-6 Lines 193-227). |
|  | -Do not put headings in your discussion, simply use a paragraph to properly explain your points in the discussion. | We have now removed subheadings in the discussion, we have only left the term conclusion to indicate that the text belongs to a different conclusive statement. |
|  | -Please, use the last paragraph and discuss the limitations of your study. The strength and future prospects this study should be discussed in a separate paragraph. | We have now repositioned the text on limitations as per suggestion (Page 16 Lines 716-721). |
|  | -I realized that there was no citation in the discussion. Despite being an empirical study, I believe there may be some articles about dementia patients and some findings that may be related to your study which can be cited as part of your discussion. | We have now discussed the findings by considering the evidence (Pages 15-16 Lines 683-706). |
| ***Reviewer 1*** | | |
| ***Title*** | Based on the title I was surprised when the abstract revealed it was a qualitative study. Could the title be rephrased to include study design and more accurately represent the paper (e.g., Experiences of increasing awareness about end of life care for those experiencing dementia: Qualitative study of carers and hospice staff). Not sure the detail about East Cheshire is needed and isn’t picked up in the abstract. | Thank you. We agree with the comment, and we have now rephrased the title into:  *‘Experiences of hospice dementia care: A qualitative study of bereaved carers and hospice clinicians.’ (Page 1 Lines 1-2).* |
| ***Abstract*** | The conclusion should be stronger (more work is always needed) – what is this study’s unique contribution? | We have now addressed this (Page 3 Lines 90-93). |
| ***Introduction*** | Nathan Davies at UCL has done quite a bit of work around decision making at the end of life for those with dementia. Although you touch on some of his work, I would bring more of his recent work in here. | We agree, we have now included more of his work in the introduction (references 4 and 15). |
|  | It would be useful to explain the differences in care between care homes and hospices for people with dementia (and therefore this justifies why hospices should be explored specifically). | We have now explained this more prominently (Page 4 Lines 136-139). |
|  | Aim is different to the one in the abstract (brings in role of the hospice and use of PCC). I would suggest removing the primary research question and have one clear aim in the abstract and end of introduction. The PRQ also only concerns carers not staff. | We have now made the aim consistent between abstract and intro. (Abstract: page 3 Lines 76-78; Intro: Page 5 Lines 174-175). |
| ***Methods*** | Would be useful to have some subheadings in the methods (e.g., participants, inclusion/exclusion criteria, data collection and data analysis). | We agree and we have now added subheadings in the methods section: Study procedure, inclusion criteria, data collection, data analysis. |
|  | Add ‘(PPEI)’ in page 4 after spelling it out. | We have now added this (Page 5 Line 186-187). |
|  | Add Participant Information Sheet on before PIS at the starts of page 5. | We have now added this (Page 5 Line 189). |
|  | Can the hospice be described in more detail to set the context (e.g., how large, is it in a deprived or affluent area, is it dementia only). | We have now included a description in the methods (Pages 5-6 Lines 193-227). |
|  | Eligibility – why did carers have to be over 50? The reason should be included. | We have removed the age limitation as in fact this was not a criterion we followed. |
|  | You’ve stated a minimum of 4 weeks had to elapsed (should be added to eligibility criteria), but was there a maximum amount of time (e.g., within the past two years)? | We have added this in the relevant text (Page 6 Lines 252-254). |
|  | The interviews with hospice staff focused on barriers and facilitators to managing people living with dementia – this is the first time this aim or focus is mentioned in the paper. It needs to come earlier and thread through. Again the interviews with the carers focused on accessing. The title states ‘increasing awareness’ and the aim is around role of the hospice and use of PCC. Perhaps there needs to be one broad aim (e.g., experiences of end-of-life care in hospices) and then several objectives underneath (e.g., role of hospice, management, barriers and facilitators). | We have now addressed this and we feel we made our argument stronger and more consistent across sections. |
|  | Add a reference for thematic analysis as there’s been many updates and different approaches. | We have now added the reference (reference 19) as our analysis was informed by Braun V, Clarke V. Reflecting on reflexive thematic analysis. Qualitative research in sport, exercise and health. 2019 Aug 8;11(4):589-97. |
|  | I would scrap the inter-reliability check and have more confidence in your qualitative analysis. I do not think qualitative data or the process behind it needs to be quantified unless there are concerns. It may be that carers and hospice staff have different views of this complex time and that would be perfectly okay. | We agree and we have now removed the relevant text. |
| ***Results*** | A table or summary of the characteristics of participants would be useful of anything other than gender and ethnicity (e.g., age, length of time since bereavement, years of experience, job role). | As recruitment occurred in only one hospice setting, we have decided at team level and with participants that no further information would be provided to ensure anonymity. |
|  | Unsure why the East Cheshire end-of-life pathway figure is in the results and discussed in the discussion – from what I can see this should be introduction or methods. This comes out of the blue. | We have now moved the text In the methods section (Pages 5-6 Lines 193-227). |
|  | Again I would lose the quant reliability check and explain how qualitatively you ensured this data was robust (e.g., in the methods include some reflexivity - have multiple professions/backgrounds looked over the themes?). Include this information about the authors. | We have now removed inter-reliability and explained more prominently the coding strategy. |
|  | Somewhere you need to address the sample size between your aim and your final number. If you had challenges recruiting then this is important to highlight and can be helpful. Also is this hospice area predominately White British or did different ethnicities decline to take part (discussing end-of-life can vary across cultures)? | We based our sample size on conceptual density (Page 7 Lines 265-267). |
|  | Results second line – should be from not form. | Thank you we have now corrected the typos (Page 7 Line 291). |
|  | The results are quite list like and could be more synthesised. Rather than relying too much on the quotes the explanation can come through from the authors and then the quotes highlight and add context/detail to the text. Rather than a list of quotes. Sometimes this is done well, but take a look through to see areas where more elaboration/synthesis can be given (e.g., Page 9 ‘2a a clinical staff member with background in GP describes her routine.’ Here I would say what the take home message is for the reader then present the quote).  Halfway down page 7, carers report different experiences and then two examples are given, what were the other three experiences? Did most have advanced care planning?  The first part of the results (1a) seems to highlight a system that is working well with joined up care (e.g., sharing of advanced care plans), working with the community. I would talk about that here. | We hope we have now been able to address this. (Please see relevant themes mentioned in the comments. |
|  | 1b is an interesting sub-theme with good interpretations. | Thank you. |
|  | A table or figure might be a good way to present the data at the start of the results. (Realised at the end there is a table – ensure this is mentioned in text although I may have just missed it). | Yes we added Table 1 in the results section. |
|  | The first half of 2b is describing how the hospice is run – its not really analysis, this needs to go further if it is to be included. The last two quotes are interesting and provide insight and discuss person centred care – treating the individual. Is there more of this in the data and perhaps this section could focus on person-centred care? We often talk about it but often we don’t know how to deliver it – staff 7 seems to get it and even staff 6 is highlighting they’d not reply on a handover sheet. | Thank you, we feel we have now improved the reporting of the analysis for theme 2b. |
|  | 2c sounds brilliant – Staff 7 quote again sounds PCC like. I’m not sure there is enough here for its own subtheme on information sharing as seems a one off point unless there’s more to it or could fit else where? | We have now condensed this subtheme with subtheme 3b (key features promoting PCC) |
|  | 2d seems to be around decision making | We have not renamed the theme 2d, however we have added some more analysis. |
|  | 6 subthemes is a lot for theme 2 – could these be synthesised together more? | Yes we condensed the subtheme 2e (carers’ expectations) with subtheme 2d (being a carer in end-of-life dementia care) as they both report on what it means being caring doe someone in end of life |
|  | 3b – what is the East Cheshire model of care? A lot of this could fit with the PCC as above. | We agree, we have now condensed the subtheme *2c information sharing* with *3b key feature of east Cheshire model of care* which is now entitled: *3b* *key features providing PPC.* |
|  | To me this is an excellently run service, and more detail about how they’re able to do this would be useful. For example, how can other services foster this environment and attitude? Perhaps goes beyond the scope of the paper but if the results could highlight this would be brilliant.  Overall less description and more synthesis and analysis. | We discussed the findings by considering the evidence to suggest directions (Pages 15-16 Lines 683-706). |
| ***Discussion*** | I would cut the second sentence. I think the introduction paragraph could be stronger. The authors found a service that used joined up care, information sharing and were delivering person-centred care and decision making at the end-of-life for people with dementia, despite a lack of funding. I would really summarise this here. | We have now added text in the first paragraph in the discussion as per suggestion and discussed the findings by considering the evidence (Pages 15-16 Lines 683-706). |
|  | The following four paragraphs of the discussion appears to be introduction of a service apart from the odd sentence which focuses on the study results. There is little reflection of the results and placing them in context with other research – in fact no references are used in the discussion at all. I would cut all the description of the service and move to intro/method and then focus here on the results and implications. | We have now moved the description of the service to the methods section (Pages 5-6 Lines 193-227). |
| ***Reviewer 2*** | | |
|  | 1. The main obstacle of the paper is that it is based on 12 interviews of less than 40 seconds each. Although it is acceptable to conduct such a study, its conclusions must be taken with caution and in the context of the place where the study was conducted. | Interviewed lasted around 40 minutes (no seconds). We have now added a bit of text explaining that study findings need to be taken with caution. (Page 16 Lines 716-717). |
|  | 2. You have mentioned that little research has been done on carers of people with dementia. However, there are several papers that explore medical staff and family caregivers of dementia patients in Israel. These studies were conducted on more than 80 interviewees and also examine home hospice care vs. inpatient care, views on treatment, and obstacles in implementing these care methods. | We have now specified that evidence is scant around the experience of the service in the UK. We appreciate that work has been done in other countries, however we feel that this would be out of scope of our investigation. We have added evidence in the discussion regarding work conducted in other countries (Pages 15-16 Lines 683-706). |
|  | 3. You have described the recruitment procedure of the interviewees in a detailed and professional manner. I would suggest adding examples of the questions they were asked. | We have now reported two examples from the topic guides, one example for carers and one for staff (Page 7 Lines 270-272; Lines 274-277). |
|  | 4. With only 12 interviewees, have you reached data saturation in the data collection process? | Yes we based our sample size on the Sample size on conceptual density (i.e., gathering data until a sufficient depth of understanding was reached), which was agreed upon by all study authors. (Page 7 Lines 265-267). |
|  | 5. You describe the process, including information sharing methods, in the Cheshire region. Is it similar to other regions in England? You also mention the Sunflower Center, and it is not clear whether this center works similarly to other day care centers. Additionally, you should elaborate more on this center and its methods of care in the introduction. | Thank you. We have added description of the service in the methods, however we do not have information about other services as the idea would be to explore with a larger project what is offered nationwide. (Pages 5-6 Lines 193-227). |
|  | 6. Regarding the barriers, you focused on the lack of resources, which is probably true. However, other studies have shown additional barriers such as a lack of knowledge and unclear regulations, among others. | Yes we agree but we reported specifically on our findings. We hope we were able to understand the comment. |
|  | 7. During the discussion, you mention the H@H rapid response and discuss the differences between this and inpatient care. I believe some of this discussion, including the presentation of these two options, should be moved to the introduction. | We have now moved the description of the service in the methods (Pages 5-6- Lines 193-227). |
|  | 8. In the discussion, the authors state that "Family carers whose loved ones stayed without specialist hospice care support (e.g., people remaining in their home without specialist end-of-life care or those admitted to hospital inpatient care) may be at increased risk of emotional turmoil and sorrows due to a lack of proper bereavement support." I do not think that you can conclude this from 12 interviews. You may hypothesize it, but I wouldn't draw a conclusion based solely on that. | We agree and we have now reworked our discussion. |
