# Supplementary material for: Experiences of hospice dementia care: A qualitative study of bereaved carers and hospice clinicians
Source: PLoS One. 2023 Nov 6;18(11):e0286493. doi: 10.1371/journal.pone.0286493 (PMC10627455; doi:10.1371/journal.pone.0286493)
Supplement: Attachment — Submitted filename: Response to reviewers.docx [file pone.0286493.s002.docx]

Table for reviewers.

| **Editor** | | |
| --- | --- | --- |
| **Where in the manuscript** | **Reviewers’ comments** | **Our response to reviewers** |
| ***Abstract*** | -This part of the abstract should be under methods since it is reflecting the number and type of participants that were recruited and interviewed “A total of 12 participants were interviewed from one service in the Northwest region in the UK. All were female and white British.” | This has now been moved as per suggestion (Abstract, methods, Page 3 Lines 83-84). |
| ***Methods*** | Nothing was mentioned with regards to “data saturation point”. Did you at any point of the interview achieve data saturation? Please state whether or not this was achieved as part of the methods. If data saturation was not achieved during the interview, kindly state it as part of the limitation of your study. | We added on conceptual density to describe that we had enough understanding of the topic after 12 interviews. We have now more promptly explained this and used the term data saturation. (Page 7 Lines 267-269). |
|  | The statement below should be part of the methods involved in recruitment of participants and should not be part of the results. Please, insert this section under methods in the appropriate location:  “Semi-structured interviews were held with 12 participants (seven clinical hospice staff and five family carers, all were female and white British) form one service in the Northwest region. Two staff were medically trained in general practice, three were nurses with one being a palliative care specialist, two were health care assistants. The interviews lasted on average 38.30 minutes.” | We have now moved the section as per suggestion (Page 7 Lines 267-272). |
| ***Results*** | -Please, do include a table that will clearly indicate the THEMES, SUBTHEMES, CODES GENERATED and REPEATITION OF CODES by participants for more clarity on the data analysis and understanding of the data at a glance. This table should be in the result section and modified as | In table 1 we have now added in the first column (highlighted text) the theme coding assigned to each theme (N). We hope this now is in agreement with the editor. |
| ***Reviewer 1*** | | |
|  | Thank you for the revised version. All my comments have been addressed sufficiently. One change I would suggest when proofing is to change the reflexive TA citation to codebook TA (as you do not discuss author background in data analysis section and outline a codebook). However I do not want to delay publication so suggest this is done at the proofing stage. | Thank you, we agree, we have now changed this in reference 19. |
| ***Reviewer 2*** | | |
|  | My minor comment is that another limitation of the study (besides the small sample, that the authors raised) is that all participants are female and white British. Patients who are men and/or not white British might have different needs. | We agree and we have now added text (Page 16 Lines 715-717). |
